# Clinical helminthiases in Thailand border regions show elevated prevalence levels using qPCR diagnostics combined with traditional microscopic methods

**DOI:** 10.1186/s13071-020-04290-0

**Published:** 2020-08-12

**Authors:** Poom Adisakwattana, Tippayarat Yoonuan, Orawan Phuphisut, Akkarin Poodeepiyasawat, Nirundorn Homsuwan, Catherine A. Gordon, Donald P. McManus, Louise E. Atkinson, Angela Mousley, Geoffrey N. Gobert

**Affiliations:** 1 Department of Helminthology, Faculty of Tropical Medicine, Mahidol University, Bangkok, 10400 Thailand; 2 Molecular Parasitology Laboratory, Infectious Diseases Division, QIMR Berghofer Medical Research Institute, Brisbane, 4006 Australia; 3 School of Biological Sciences, Queenʼs University Belfast, Belfast, BT9 5DL UK

**Keywords:** Helminthiases, Kato-Katz, molecular diagnostics, qPCR, Thailand border regions, Southeast Asia

## Abstract

**Background:**

Under-regulated national borders in Southeast Asia represent potential regions for enhanced parasitic helminth transmission and present barriers to helminthiasis disease control.

**Methods:**

Three Thailand border regions close to Myanmar, Laos and Cambodia were surveyed for clinical parasitic helminth disease. In-field microscopy was performed on stools from 567 individuals. Sub-samples were transported to Bangkok for molecular analysis comprising three multiplex qPCR assays.

**Results:**

The overall helminth infection prevalence was 17.99% as assessed by Kato-Katz and 24.51% by qPCR. The combined prevalence of the two methods was 28.57%; the most predominant species detected were *Opisthorchis viverrini* (18.34%), hookworm (6.88%; *Ancylostoma* spp. and *Necator americanus*), *Ascaris lumbricoides* (2.29%) and *Trichuris trichiura* (1.76%).

**Conclusions:**

These data demonstrate the value of molecular diagnostics for determining more precise prevalence levels of helminthiases in Southeast Asia. Availability of such accurate prevalence information will help guide future public health initiatives and highlights the need for more rigorous surveillance and timely intervention in these regions.
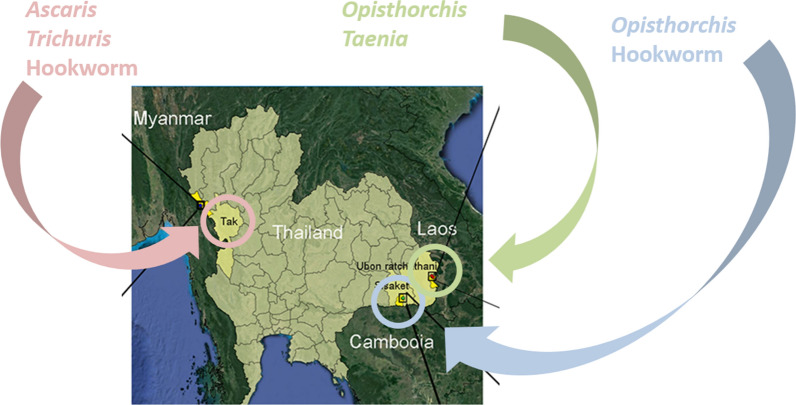

## Background

Parasitic worm infections (helminthiases) of humans greatly impact low to medium income countries, including Thailand and bordering countries. Worldwide, helminths infect an estimated 1.45 billion people [1], with one third of cases occurring in Southeast Asia [2]. Most affected are young people, particularly pre- and primary school-aged children (0–12 years), leading to a range of health issues including stunted growth and delayed mental development [3]. Since most helminth diseases cause chronic morbidity (95% of health-attributed losses) rather than acute disease, the impact on the health and economic output of endemic areas is frequently reported in terms of disability-adjusted life years (DALYS); these measure the years lost due to morbidity, illness, and premature death. Helminth infections worldwide in 2010 were responsible for 14 million DALYs [4]. Hookworm infection alone, largely due to anaemia, was responsible for over 4 million DALYs, with the total economic loss due to reduced productivity estimated to be between US$7.5–138.9 billion [5].

Helminth infections are routinely field diagnosed using the “gold standard” Kato-Katz (KK) method, a low-sensitivity microscopic technique, which is limited by species range and quantitative accuracy. The KK can underestimate prevalence [6] and therefore impact on MDA (mass drug administration) programmes and outcomes.

The previous national surveillance on intestinal helminthiases in Thailand, conducted by the Thai Ministry of Public Health in 2009, revealed an overall prevalence (among 15,555 Thai people) of 18.1% with the highest prevalence in the North-eastern region of Thailand [7]. Recently, only limited spatial and temporal surveillance has been conducted, which may be insufficient to adequately guide the national public health initiatives [8–10]. Moreover, helminth prevalence surveillance studies carried out to date [7, 9, 11] have been undertaken primarily using microscopy-based faecal examination (KK) without combining with molecular detection, which may underestimate the actual extent of helminth infection.

In this study we explore the utility of molecular diagnostic procedures for the detection of active helminth disease in Thai populations close to border regions with Myanmar, Laos and Cambodia. Comparisons with the traditional KK microscopy are made and the strengths and weakness of both methods assessed and discussed. The central aim of this work was to provide a more accurate picture of the prevalence of helminthiases in these areas. With this key information public health officials will be able to better plan control measures for the future to reduce helminth disease transmission.

## Methods

### Study areas

**Fig. 1:**
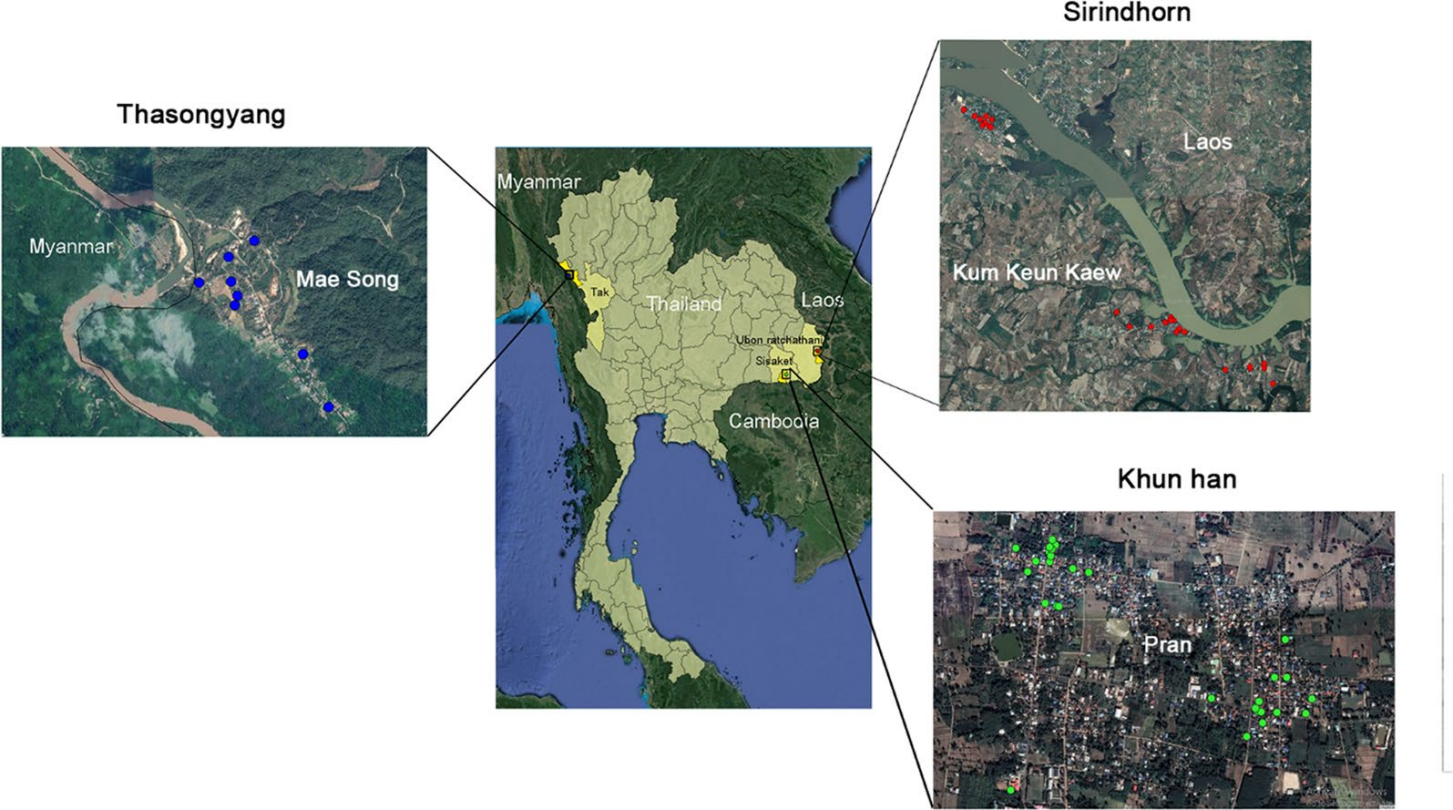
Location of field sites. The Thai-Myanmar (Tak-Thasongyang) with the Moei River, Thai-Lao (Ubon Ratchathani-Sirindhorn) with the Mekong River, and Thai-Cambodia (Sisaket- Khun Han) border regions are shown. Specific field collections sites are marked for the three regions in the enlargements

All study sites were located near three Thai border regions Villages within these border regions were surveyed (Fig. 1): (i) the Mae Song Sub-District, Thasongyang District and Tak Province close to the Thai-Myanmar border; (ii) Kham Khuean Kaeo Sub-District of Sirindhorn District within Ubon Ratchathani Province at the Thai-Lao border; and (iii) the Phran Sub-District of Khun Han District in Sisaket Province at close proximity to the Thai-Cambodia border. Based on annual income per person per year, the regions surveyed in Tak Province are considered to be of low socioeconomic status, while those in Srisaket and Ubonratchathani Provinces are considered to be of middle socioeconomic status. All study sites were located within Thailand but were close (1–10 km) to the bordering countries. The three sites included multiple villages within the provinces of Sisaket, Tak and Ubon Ratchathani (Fig. 1). The spots marked in the enlarged panels in the Fig. 1 represent individual houses normally located in clusters and distribute across several village. Within these provinces a total of 14 villages were surveyed (7, 1 and 6, respectively). The results from the two diagnostic approaches have been reported to health promoting hospitals in the targeted areas for further action by local medical doctors or heath officers according to treatment guidelines provided by the Department of Disease Control, Ministry of Public Health, Thailand [12].

### Sample collection

The three field sites were visited on one occasion each between December 2017 and February 2018. In the 14 villages surveyed, 567 clinical faecal samples were collected. Across the Sisaket and Ubon Ratchathani sites 200 faecal samples from both provinces were collected (400 in total) and 167 were collected from the Tak site. Ages of participants (311 females; 256 males) ranged from 1.5 to 88 years; age demographic data were not collected for 43 sampled individuals.

Labelled sample cups were provided to health volunteers for distribution to participants. The samples were collected the next day either by health volunteers or brought to a collection point by participants. Information on age, gender, occupation, and geographical location, was collected at the same time as faecal samples. KK analysis was conducted at the site. Parallel faecal samples were stored in 80% (v/v) ethanol for transport at ambient temperature to Bangkok for molecular analysis.

KK was performed on all human faecal samples collected as previously reported [13–15]. In brief, individual stool samples were pressed through a stainless sieve (size 40 mesh: 420 µm sieve opening), and the non-retained material used to fill a kit template which equated to 39.2 mg of material. Glycerin-malachite green-soaked cellophane was placed on the sample with a glass slide and firmly pressed to spread the stool evenly across the surface. The slide was viewed on a light microscope after 30 min. Two slides were prepared from each sample and analysed independently by two trained microscopists. All eggs were identified as observed when possible. All samples were anonymised and analysed blinded.

### Multiplex qPCR

Due to the breadth of the potential helminth infection profile amongst the participants, this study screened for 9 helminth species. As a result, three separate multiplex PCR assays were developed to identify the presence of helminth DNA in faecal samples. Faecal DNA was isolated using QIAamp^®^ Fast DNA Stool Mini Kit (Qiagen GmbH, Hilden, Germany). A 1% agarose/TAE electrophoresis gel was used to check all samples after faecal DNA purification, all samples showed high molecular weight DNA after treatment with RNase A. Positive controls were prepared by isolation of genomic DNA from adult parasites using QIAamp^®^ DNA Mini Kits (Qiagen GmbH). Each assay was designed to detect three different helminth species simultaneously as follows: Assay 1: *Ascaris lumbricoides*, *Strongyloides stercoralis*, *Trichuris trichiura*; Assay 2: *Ancylostoma* spp., *Necator americanus*, *Opisthorchis viverrini*; Assay 3: *Taenia solium*, *T. saginata*, *Schistosoma japonicum*/*S.* *mekongi*. Primers and probes used in each assay are presented in Additional file 1: Table S1 [16–19]. Multiplex qPCR was performed in duplicate in a final volume of 20 µl by mixing 1 µl of faecal DNA (100 ng/µl) with 10 µl of iQ Multiplex Powermix (Bio-Rad Laboratories Inc., Hercules, CA, USA) and 200 nM each of forward (Fw) and reverse (Rv) primers in addition to 100 nM of the appropriate TaqMan probe. Amplification was performed using the a CFX96 Real-Time PCR System (Bio-Rad Laboratories, Hercules, CA) with pre-incubation at 95 °C for 2 min, followed by 40 cycles of 95 °C for 10 s and 60 °C for 30 s. All positive control samples and a subset of clinical samples were checked for assay specificity by sequencing amplicons to confirm the identity of the helminth species detected.

### Statistical analyses

Excel (Microsoft) and SAS software (SAS Institute) were employed for statistical analysis. A sample was considered positive if there was at least one egg on a KK slide, or if a positive quantification cycle (Cq) score was generated by qPCR (Cq > 37 was considered negative, Cq < 37 was considered positive). For the KK, egg counts were transformed to eggs per gram of faeces (EPG) by multiplying the average egg count from all slides by 25.5, based on the amount of material retained and examined after processing. Geometric mean EPG (GMEPG) was calculated using log_10_-transformed egg counts [20]. Standard formulae were used to calculate 95% confidence intervals (CI) for prevalence and intensity using biomodal distribution (prevalence) and the lognormal distribution (infection intensity). Relative sensitivity and specificity were calculated in two ways: (i) by combining the results of both KK and qPCR to act as the reference standard; and (ii) by using the qPCR results as the reference standard to calculate sensitivity and specificity of the KK, and the KK as the reference standard to calculate sensitivity and specificity of qPCR. Significance (Chi square, *P*-value) was calculated using general estimating equations in SAS; *P* ≤ 0.05 was considered significant. The kappa coefficient was calculated to show agreement between the KK and qPCR methods. A coefficient between 0.81–1.00 was considered as almost perfect agreement, 0.61–0.80 high, 0.41–0.60 moderate, and 0.01–0.40 as low agreement.

## Results

A summary of individual participant demographics within the three field-site cohorts, as well as the KK and qPCR results for each participant are presented in Additional file 2: Table S2.

### Kato-Katz field-based analyses

KK analyses of all faecal samples collected from the three field sites indicated an overall prevalence of any helminth infection in all individuals of 17.99% (95% CI: 14.82–21.16%). The most common parasitic worms detected were *O. viverrini* (8.82%; 95% CI: 6.48–11.16%), *A. lumbricoides* (2.12%; 95% CI: 0.93–3.30%), *T. trichiura* (1.59%; 95% CI: 0.56–2.62%), *Taenia* spp. (1.23%; 95% CI: 0.03–2.15%) and hookworm (*Ancylostoma* spp. and/or *N. americanus*; 5.82%; 95% CI: 3.89–7.75%) (Table 1). Hookworm infection was recorded based on the identification of the eggs of either hookworm species since definitive species-level diagnosis is challenging by microscopy. There were no statistical differences in helminth prevalence based on age, gender, or geographical location. No indication of active *S. mekongi* or *S. japonicum* infections were evident by KK analysis in any of the samples collected, across the three field sites. Intensity of infection was calculated by KK as the GMEPG. The highest GMEPG was evident with *A. lumbricoides* (2595.6) followed by *Taenia* spp. (1009.19) (Table 1).

**Table 1:** Summary of prevalence and intensity levels for helminth species determined by Kato-Katz and qPCR combined for all three regions surveyed

	Kato-Katz							qPCR			
	No. positive^a^	Prevalence (%)	95% CI	EPG	95% CI	GMEPG	95% CI		No. positive^a^	Prevalence (%)	95% CI
Positive any species	102	17.99	14.82–21.16					Positive any species	139	24.51	20.96–28.07
*O. viverrini*	50	8.82	6.48–11.16	11.52	5.29–17.74	65.49	48.28–88.83	*O. viverrini*	97	17.28	14.16–20.41
Hookworm	33	5.82	3.89–7.75	12.55	3.78–21.33	83.83	53.42–121.54	*N. americanus*	13	2.29	1.06–3.53
								*Ancylostoma* spp.	9	1.59	0.56–2.62
*A. lumbricoides*	12	2.12	0.93–3.30	313.8	0–632.24	2595.6	452.83–14878.05	*A. lumbricoides*	12	2.12	0.93–3.30
*T. trichiura*	9	1.59	0.56–2.62	11.16	0–30.30	92.87	22.23–387.90	*T. trichiura*	2	0.35	0–0.84
*Taenia* sp.	7	1.23	0.03–2.15	71.45	0–149.33	1009.19	73.35–13884.48	*T. saginata*	6	1.06	0.21–1.90
								*T. solium*	1	0.18	0–0.52
*E. vermicularis*	4	0.71	0–1.40	0.4	0–0.96	39.93	9.60–166.09				
*Trichostrongyloides*	1	0.18	0–0.52	0.044	0–0.133	25.51	na				
								*S. stercoralis*	7	1.23	0.03–2.15

^a^From 567 samples

*Abbreviations*: EPG, arithmetic EPG; 95% CI, 95% confidence interval; na, not available

### Molecular diagnostics

Stool samples were transported to Mahidol University, Bangkok, for processing and molecular analysis. A panel of three separate multiplex qPCR assays was applied to all samples (*n* = 567). When qPCR was used independently, the overall prevalence (for any helminth) was higher to that obtained by the KK method (24.51%; 95% CI: 20.96–28.07%; Table 1). Major helminth species identified were: *O. viverrini* (17.28%; 95% CI: 14.16–20.41%), *A. lumbricoides* (2.12%; 95% CI: 0.93–3.30%), hookworm designated as either *Ancylostoma* spp. (1.59%; 95% CI: 0.56–2.62%) or *N. americanus* (2.29%; 95% CI: 1.06–3.53%) and *T. trichiura* (1.59%; 95% CI: 0.56–2.62%). All qPCRs were negative for *S. mekongi* and *S. japonicum* for all samples collected across the three field sites surveyed.

The range of Cq scores for each helminth species detected, compared to the egg burdens determined by KK, is presented in Additional file 3: Table S3. Cq cut-off values were based on egg intensity (EPG). For *O. viverrini,* detected by KK in the largest number of samples (*n* = 38), the Cq range presented for lower egg numbers (1–5) was 22.63–32.09 and for higher egg numbers (> 5) was 21.93–25.19. Other parasite species, despite having smaller positive sample numbers, presented similar differentiation of Cq ranges between lower and higher egg burdens (Additional file 3: Table S3).

The kappa coefficient showed high levels of agreement between the qPCR and KK for *A. lumbricoides* (κ = 0.91), and *Taenia* spp. (κ = 0.86), and moderate agreement for *O. viverrini* (κ = 0.54) and hookworm spp. (κ = 0.53), while *T. trichiura* showed low agreement (κ = 0.18) (Table 2).

**Table 2:** Kappa analysis for agreement of the qPCR *vs* Kato-Katz methods

	No. positive Kato-Katz^a^	No. positive qPCR^a^	Kappa
*T. trichiura*	9	2	0.18
Hookworm^b^	33	23	0.53
*O. viverrini*	50	97	0.54
*Taenia*^c^	7	7	0.86
*A. lumbricoides*	12	12	0.91

^a^From 567 samples

^b^qPCR results from *Ancylostoma* spp. and *N. americanus* probes

^c^qPCR results from *T. solium* and *T. saginata* probes

### Complementation of methods and regional differences

**Fig. 2:**
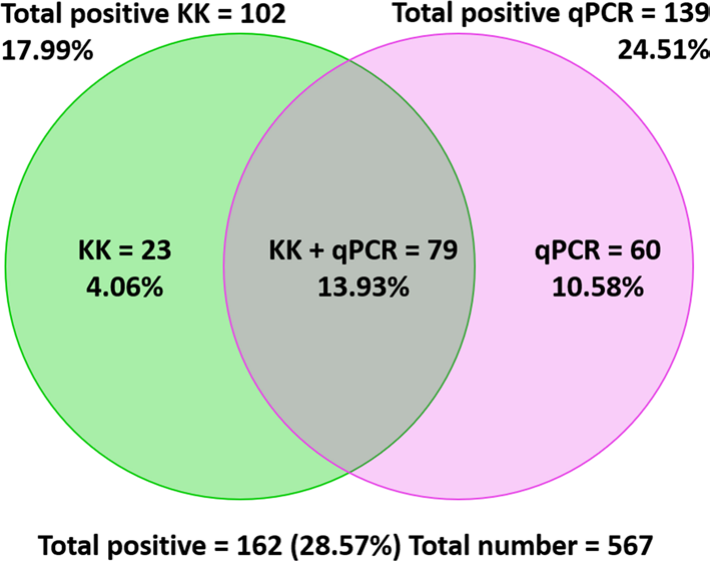
Venn diagram demonstrating the overall prevalence and agreement between the Kato-Katz and qPCR diagnostic methods used in this study

Results from the KK and qPCR were combined for further prevalence analysis and considered across the three field sites (Table 3). The overall prevalence by KK and qPCR combined was 28.57% (95% CI: 24.84–32.30%). The overall prevalence and agreement between the two methods is presented in Fig. 2. As shown in Table 3 with both methods, the most dominant species were *O. viverrini* (18.34%; 95% CI: 15.15–21.54%), hookworm (*Ancylostoma* spp. or *N. americanus*, 6.88%; 95% CI: 4.79–8.97%), *A. lumbricoides* (2.29%; 95% CI: 1.06–3.53%), *T. trichiura* (1.76%; 95% CI: 0.68–2.85%) and *Taenia* spp. (1.41%; 95% CI: 0.44–2.38).

**Table 3:** Helminth species detected using combined Kato-Katz and qPCR data for the three border regions collectively

	Number^a^	Prevalence (%)	95% CI
Positive by Kato-Katz only	102	17.99	14.82–21.16
Positive by qPCR only	139	24.51	20.96–28.07
Positive by Kato-Katz or qPCR	162	28.57	24.84–32.30
Negative all species	405	71.43	67.70–75.16
*A. lumbricoides*	13	2.29	1.06–3.53
*T. trichiura*	10	1.76	0.68–2.85
*Trichostrongyloides*	1	0.18	0–0.52
*E. vermicularis*	4	0.71	0–1.40
*O. viverrini*	104	18.34	15.15–21.54
Hookworm	39	6.88	4.79–8.97
*Taenia*	8	1.41	0.44–2.38
*S. stercoralis*	7	1.23	0.03–2.15
*A. lumbricoides* only	2	0.35	0–0.84
*T. trichiura* only	4	0.71	0–1.40
*Trichostrongyloides* only	1	0.18	0–0.52
*E. vermicularis* only	0	na	na
*O. viverrini* only	95	16.75	13.67–19.84
Hookworm only	29	5.11	3.30–6.93
*Taenia* only	3	0.53	0–1.13
*S. stercoralis* only	5	0.88	0.11–0.65
*A. lumbricoides* + *T. trichiura*	1	0.18	0–0.52
*A. lumbricoides* + *E. vermicularis*	2	0.35	0–0.84
*A. lumbricoides* + Hookworm	6	1.06	0.21–1.90
*A. lumbricoides* + *S. stercoralis*	1	0.18	0–0.52
*T. trichiura* + *O. viverrini*	2	0.35	0–0.84
*T. trichiura* + Hookworm	2	0.35	0–0.84
*T. trichiura* + *S. stercoralis*	1	0.18	0–0.52
*E. vermicularis* + *O. viverrini*	1	0.18	0–0.52
*E. vermicularis* + *S. stercoralis*	1	0.18	0–0.52
*O. viverrini* + Hookworm	1	0.18	0–0.52
*O. viverrini* + *Taenia*	4	0.71	0–1.40
Hookworm + *O. viverrini* + *A. lumbricoides*	1	0.18	0–0.52

^a^From 567 samples

*Abbreviation*: na, not available

Using the KK and qPCR results combined, but considering the three field sites separately (Table 4), there was an overall prevalence of any helminth species of 28.14% in Tak, 31.00% in Ubon Ratchathani and 26.50% in Sisaket. Using the KK/qPCR combined dataset the most prevalent human helminthiases in the three individual regions surveyed were: for Tak, (Myanmar border); hookworm (17.96%; 95% CI: 12.08–23.85%), *A. lumbricoides* (7.78%; 95% CI: 3.68–11.89%) and *T. trichiura* (4.79%; 95% CI: 1.52–8.06%); for Ubon Ratchathani (Lao border): *O. viverrini* (28.00%; 95% CI: 21.72–34.28%) and *Taenia* (3.50%; 95% CI: 0.93–6.07%); and for Sisaket (Cambodia border): *O. viverrini* (22.00%; 95% CI: 16.21–27.79%) and hookworm (4.00%; 95% CI: 1.26–6.74%). A complete summary of results by region is shown in Additional file 4: Table S4.

**Table 4:** Prevalence of helminthiases for each of the three regions surveyed using the combined Kato-Katz and qPCR data

	Tak			Ubon Ratchathani			Sisaket		
	Number^**a**^	Prevalence (%)	95% CI	Number ^b^	Prevalence (%)	95% CI	Number ^c^	Prevalence (%)	95% CI
Positive any species	47	28.14	21.25–35.03	62	31.00	24.53–37.47	53	26.50	20.33–32.67
Negative all species	120	71.86	64.97–78.75	138	69.00	62.52–75.47	147	73.50	67.33–79.67
*A. lumbricoides*	13	**7.78**	3.68–11.89	0	na	na	0	na	na
*T. trichiura*	8	**4.79**	1.52–8.06	2	1.00	0–2.39	0	na	na
*Trichostrongyloides*	1	0.60	0–1.79	0	na	na	0	na	na
*E. vermicularis*	2	1.20	0–2.86	2	1.00	0–2.39	0	na	na
*O. viverrini*	4	2.40	0–4.74	56	**28.00**	21.72–34.28	44	**22.00**	16.21–27.79
Hookworm	30	**17.96**	12.08–23.85	1	0.50	0–1.49	8	**4.00**	1.26–6.74
*Taenia* spp.	1	0.60	0–1.78	7	**3.50**	0.93–6.07	0	na	na
*S. stercoralis*	3	1.80	0–3.83	2	1.00	0–2.39	2	1.00	0–2.39

*Note*: The highest prevalence rates species for each region are given in bold

^a^From 167 samples

^b^From 200 samples

^c^From 200 samples

*Abbreviation*: na, not available

There was a relatively low level of polyparasitism in all three regions surveyed, with 3.9% of samples presenting as a dual infection. As shown in Table 3, the only co-infections, as determined by KK or qPCR, consisting of two or more helminth species (in more than 1 sample) included: *A. lumbricoides* and hookworm (6 cases, prevalence 1.06%; 95% CI: 0.21–1.90%); *O. viverrini* and *Taenia* (4 cases, prevalence 0.71%; 95% CI: 0–1.4%); *T. trichiura* and *O. viverrini* or hookworm (both 2 cases each, prevalence 0.35%; 95% CI: 0–0.84%).

## Discussion

Infection with *O. viverrini* was by far the most common species detected in this study, as shown by both the KK procedure (8.82%) and qPCR analysis (17.28%); when KK/qPCR was combined the prevalence of *O. viverrini* was 18.34% (see Tables 1 and 3). In 2009, the national prevalence of *O. viverrini* in Thailand was reported as 8.7% based on a modified KK [7]. *Opisthorchis viverrini* is a food-borne trematode fluke which is transmitted by consumption of raw or undercooked fish. Koi, a dish of raw, spiced fish, is a popular dish in Thailand and Lao, providing a ready means of infection with this parasite; indeed, infection with *O. viverrini* is relatively common in Thailand, Lao, Myanmar and Cambodia [21]. In addition, due to the consumption of Koi and other raw fish dishes, cholangiocarcinoma, which is induced by *O. viverrini*, is a major health problem [2, 7, 11, 22].

The prevalence of the soil-transmitted helminths (STH; hookworm, *T. trichiura*, *A. lumbricoides, S. stercoralis*), and *Taenia* spp. was low; hookworm was the most prevalent at 5.82% (by KK, see Table 1), followed by *A. lumbricoides*, *S. stercoralis* and *Taenia* spp. STH are endemic in Thailand, including zoonotic forms of *Trichuris* (*T. vulpis*) and the hookworm *A. ceylanicum* [23]. Improved detection of hookworm species was not apparent using qPCR (Table 1). Primers used in the present study did not differentiate between *A. duodenale* and *A. ceylanicum*, and it is therefore possible that this important zoonotic species also exists in the study areas [7]. In previous surveys of pre-school- and school-aged children at the Thailand-Myanmar border, the prevalence of STHs was > 20% in all pre-school centres and primary schools (data from Thailand Development of Children in Remote and Poverty Area Project, Ministry of Public Health, Thailand (http://www.psproject.org/News_propagandise.htm, in Thai). As indicated earlier, no differences in infection prevalence by age were evident in our study, but within the cohort of 567 individuals, only 52 were under 12 years of age.

Kappa analysis showed the best agreement between the qPCR and KK methods was 0.91 for *A. lumbricoides*, indicating concordance between the two diagnostic approaches, followed by 0.86 for *Taenia* spp. (Table 2). The lowest kappa score, demonstrating the least agreement between the two tests, was for *T. trichiura* (0.18; Table 2), but the overall number of positive samples for *T. trichiura* (9 by KK, and 2 by qPCR) was low, making it difficult to draw robust conclusions from these data. One possible reason for the low number of *T. trichiura* infections positive by qPCR relates to the thickness of the *T. trichiura* egg-shell, as a result of which complete lysis of the egg and DNA extraction are unlikely to be successful [24]. In previous studies the addition of bead homogenisation prior to DNA extraction has been utilised to effectively extract DNA from this species [24]. The addition of this step may also increase the availability of DNA from other helminth species present in the samples, potentially reducing the number of false negatives. Further, with respect to false negatives, the addition of a spike-in control could be considered however this would require a qPCR detection channel which would greatly reduce the throughput capacity of the assay. A spike-in control could be used intermittently as a quality control to demonstrate genomic DNA isolation capacity.

There was a low concordance (0.54) between the two diagnostic tests for *O. viverrini*, indicating only moderate agreement between the two. The qPCR identified nearly twice as many positive samples as the KK, accounting for much of the lack of agreement evident. The KK is known to lack sensitivity, particularly in low intensity infections, [25, 26] and likely accounts for the lower prevalence of *O. viverrini* determined in this study by this method. Furthermore, the GMEPG for *O. viverrini* was a relatively low 65.49 indicating that the KK missed low-grade infections that were subsequently identified by qPCR (Table 1).

Overall, the data presented here for Thai border communities highlight the value of molecular diagnostic tools for regional assessment of helminth prevalence in Southeast Asia as these resources provide accurate quantitative prevalence figures for helminthiases. In the Tak region bordering Myanmar, *Ascaris*, *Trichuris* and hookworm were all prominent parasite present. The absence of *Opisthorchis* in Tak was contrasted by high levels of this fluke in Ubon (Laos border) and Sisaket (Cambodia border) which are both in the northeast of the country (Table 4). In the Tak region, especially the Thasongyang District, the area is predominantly rural, mountainous and remote. The people live in poverty with poor hygiene, poor sanitation, lack of education and inadequate public utilities. Low quality/lack of latrines and a lack of water drives the open defecation behaviour of people in this region which directly impacts STH transmission. In contrast, in the northeast of the country the Ubon Ratchathani and Sisaket regions are urbanized with adequate public utilities however, eating of raw fish is still commonplace resulting in higher *Opisthorchis* infections in this region. Raw-fish-eating behaviour is not observed in the Tak region.

Molecular methods have become increasingly common, particularly multiplex approaches, to identify a wide range of pathogens as many helminth and protozoan parasites overlap in endemic regions [22, 27–29]. The correlation of Cq scores and egg numbers was performed in this study but could not differentiate beyond low and higher egg burdens, an issue which has been discussed by others previously [30]. The use of molecular diagnostics to obtain a more complete picture of helminthiases in Southeast Asia has proven highly effective in the Philippines where much higher levels of polyparasitism are evident and where the overall prevalence of *S. japonicum* (~91%), *A. lumbricoides* (58.17%), *T. saginata* (42.57%) and *A. duodenale* (48.07%) determined by qPCR was substantially higher than by the KK [28, 31]. The data presented here provide a contrasting profile of local helminthiases in the three Thai border regions surveyed compared to Palapag in the Philippines [28, 31], with the presence of *O. viverrini* and absence of active schistosomiasis being key differences. In addition, compared with the Philippines Palapag study site, we recorded low levels of polyparasitism (3.9% of samples were double co-infections). A 2018 study in central Thailand reported low prevalence of *S. stercoralis* (1.5%) and *O. viverrini* (0.4%) in children using both a simple wet mount smear and the formalin-ether concentration method [10]. Another study in Chachoengsao Province, central Thailand, using a simple direct smear and formalin ethyl acetate concentration technique with parasite identification in positive samples confirmed using microscopy, reported an overall prevalence of 16.1% intestinal parasitic infections; STH (14.3%) were more common than protozoan infections (1.8%), and the most common intestinal parasites were hookworms (6.7%), then *S. stercoralis*, (5.0%), *A. lumbricoides* (1.3%) and *T. trichiura* (1.3%) [9]. The findings we present here suggest that both of these studies are likely to have underestimated the prevalence of helminthiases in these two areas by not using KK and qPCR in combination. A 2016 review by Dunn et al. [32] noted that KK is the most commonly used method for the detection of STH in SEA [32]. Their metanalysis noted that in Thailand, from 1953–2015, 20 of 55 studies used KK, and found STH for hookworm, *Ascaris* and *Trichuris* as the most prevalent infections [32].

## Conclusions

The prevalence of helminthic infections in communities of Thailand-Myanmar, Thailand-Laos and Thailand-Cambodia border regions was higher by qPCR analysis compared with previous reports based on microscopy methods alone. As a consequence, more comprehensive surveys of helminth infection prevalence and intensity using molecular methods are urgently required in these areas and should be coupled with more widespread public awareness of helminth diseases and the instigation of health educational interventions.

## Supplementary information

**Additional file 1: Table S1.** Primers used for the three multiplex quantitative PCR assays.

**Additional file 2: Table S2.** Complete collection parameters and diagnostic results for the Kato-Katz and qPCR for the three field sites.

**Additional file 3: Table S3.** Range of Cq scores for qPCR stratified based on egg burdens determined by the Kato-Katz counts.

**Additional file 4: Table S4.** Helminth species detected using the combined Kato-Katz and qPCR data; each of the three regions is presented separately.

## Data Availability

All data generated or analysed during this study are included in this published article, and its additional files.
